# Differences in the Pathogenicity of the p.H723R Mutation of the Common Deafness-Associated *SLC26A4* Gene in Humans and Mice

**DOI:** 10.1371/journal.pone.0064906

**Published:** 2013-06-03

**Authors:** Ying-Chang Lu, Chen-Chi Wu, Ting-Hua Yang, Yin-Hung Lin, I-Shing Yu, Shu-Wha Lin, Qing Chang, Xi Lin, Jau-Min Wong, Chuan-Jen Hsu

**Affiliations:** 1 Institute of Biomedical Engineering, National Taiwan University, Taipei, Taiwan; 2 Department of Otolaryngology, National Taiwan University Hospital, Taipei, Taiwan; 3 Department of Medical Genetics, National Taiwan University Hospital, Taipei, Taiwan; 4 Transgenic Mouse Models Core (TMMC), Division of Genomic Medicine, Research Center For Medical Excellence, National Taiwan University, Taipei, Taiwan; 5 Department of Otolaryngology, Emory University School of Medicine, Atlanta, Georgia, United States of America; 6 Department of Otolaryngology, College of Medicine, National Taiwan University, Taipei, Taiwan; University of Texas Health Science Center, United States of America

## Abstract

Mutations in the *SLC26A4* gene are a common cause of human hereditary hearing impairment worldwide. Previous studies have demonstrated that different *SLC26A4* mutations have different pathogenetic mechanisms. By using a genotype-driven approach, we established a knock-in mouse model (i.e., *Slc26a4^tm2Dontuh/tm2Dontuh^* mice) homozygous for the common p.H723R mutation in the East Asian population. To verify the pathogenicity of the p.H723R allele in mice, we further generated mice with compound heterozygous mutations (i.e., *Slc26a4^tm1Dontuh/tm2Dontuh^*) by intercrossing *Slc26a4^+/tm2Dontuh^* mice with *Slc26a4^tm1Dontuh/tm1Dontuh^* mice, which segregated the c.919-2A>G mutation with an abolished *Slc26a4* function. Mice were then subjected to audiologic assessments, a battery of vestibular evaluations, inner ear morphological studies, and noise exposure experiments. The results were unexpected; both *Slc26a4^tm2Dontuh/tm2Dontuh^* and *Slc26a4^tm1Dontuh/tm2Dontuh^* mice showed normal audiovestibular phenotypes and inner ear morphology, and they did not show significantly higher shifts in hearing thresholds after noise exposure than the wild-type mice. The results indicated not only the p.H723R allele was non-pathogenic in mice, but also a single p.H723R allele was sufficient to maintain normal inner ear physiology in heterozygous compound mice. There might be discrepancies in the pathogenicity of specific *SLC26A4* mutations in humans and mice; therefore, precautions should be taken when extrapolating the results of animal studies to humans.

## Introduction

Mutations in the *SLC26A4* (*PDS*, GeneID: 5172) gene are the second most frequent cause of human hereditary hearing impairment worldwide, next to mutations in the *GJB2* (GeneID: 2706) gene [1]. In some populations, *SLC26A4* mutations can be identified in approximately 13–14% of deaf patients [2]. *SLC26A4* encodes pendrin, an iodide/chloride/bicarbonate transporter expressed in the inner ear [3], thyroid [4], kidney [5], [6], salivary duct [7], and respiratory tract [8]. Recessive *SLC26A4* mutations contribute to both Pendred syndrome (PS; MIM #274600) [9] and nonsyndromic hearing loss (DFNB4; MIM #600791) [10], which share the phenotypes of sensorineural hearing impairment (SNHI) accompanied by an enlarged vestibular aqueduct (EVA; MIM 603545) and/or incomplete partition of the cochlea (i.e., Mondini dysplasia), although the phenotype of PS also includes goiter. To date, more than 100 *SLC26A4* mutations have been identified (Pendred/BOR Homepage; www.healthcare.uiowa.edu/labs/pendredandbor). Previous reports have described that different *SLC26A4* genotypes were correlated with distinct clinical phenotypes, and patients with PS are more likely to have 2 *SLC26A4* mutant alleles than those with DFNB4 [11], [12]. Many affected patients suffer from progressive or fluctuating hearing loss [13], implying that the natural course can be halted with preventive or therapeutic measures if the pathogenetic mechanisms of *SLC26A4* mutations are better elucidated.

In recent years, the understanding of the pathogenesis of DFNB4 and PS has been accelerated by various mouse models with mutations in the *Slc26a4* (GeneID: 23985) gene. Certain mouse models revealed congenital profound hearing loss, including the knock-out *Slc26a4^−/−^* mice [14], the *Slc26a4^loop/loop^* mice with the p.S408F mutation [15], and the *Slc26a4^tm1Dontuh/tm1Dontuh^* mice with the c.919-A>G mutation that we previously reported [16]. The conditional knock-out Tg[E];Tg[R];*Slc26a4* ^Δ*/*Δ^ mice demonstrated hearing loss of various severity dependent on the time of *Slc26a4* expression, with doxycycline initiation at E18.5 resulting in partial hearing loss [17]. These mouse models have provided excellent insight into the pathogenesis; however, 2 basic problems might hurdle the bench-to-bedside translation. First, similar to their human counterparts, mice with different mutations, to some extent, demonstrated different phenotypes, indicating that the pathology associated with each distinct mutation is different. Second, to date, no mouse model that can perfectly simulate the progressive or fluctuating hearing loss in humans has been reported. Investigating mice with other *Slc26a4* mutations might tackle these problems. Accordingly, in this study, we generated a knock-in mouse model with the p.H723R (c.2168A>G) mutation, a common *SLC26A4* mutation in the East Asian population [18]–[22], and then, we characterized the associated audiovestibular phenotypes as well as the inner ear pathology.

## Materials and Methods

### Construction of Slc26a4^tm2Dontuh/tm2Dontuh^ Knock-in Mice

**Figure 1:**
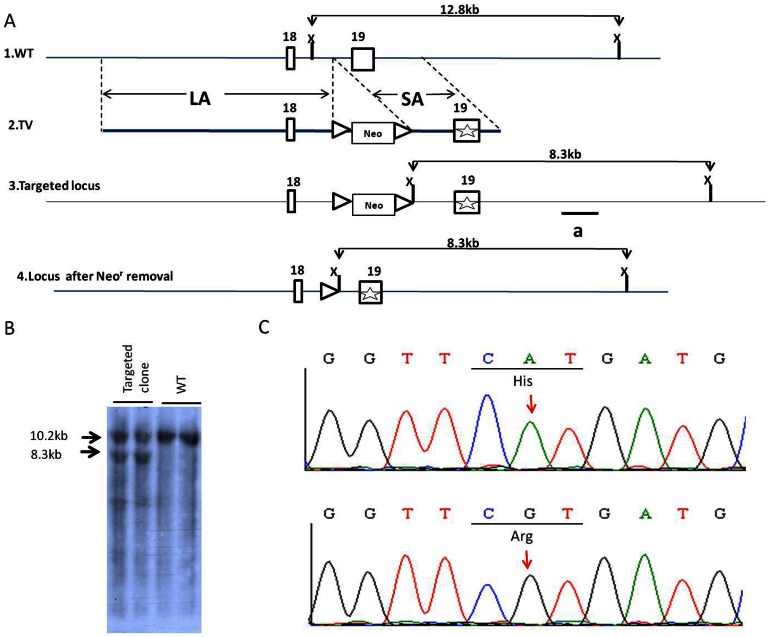
Generation of mice with the *Slc26a4* p.H723R mutation. (A) Targeting scheme. A BAC clone (clone no. bMQ-323G13 Geneservice™) from the 129S7/AB2.2 BAC library containing the mouse *Slc26a4* genomic region was used to construct the targeting vector. (1) Restriction map of the wild-type genomic mouse *Slc26a4* locus. The expected size of the *Xba*I restriction fragment was 12.8 kb. (2) Targeting vector (TV) construction. The *loxP*-flanked neomycin resistance gene (*neo*) was used as a selection marker during embryonic stem (ES) cell culture. The c.2168 A>G mutation in exon 19 is labeled with a star. (3–4) The targeted locus was introduced, and then, the *neo* cassette was removed. LA, long arm; SA, short arm. (B) Southern blot analysis of ES cell clones. Genomic DNA from 2 targeted and 2 wild-type clones were digested with *Xba*I and hybridized with the probe to verify the targeting event. (C) DNA sequencing of *Slc26a4^+/+^* and *Slc26a4^ tm2Dontuh/tm2Dontuh^* mice. The electrophoretogram shows the p.H723R mutation. The A to G mutation (arrow) at position 2168 led to the replacement of a histidine (His, H) residue at position 723 with arginine (Arg, R).

The mutation gene-targeting vector was constructed using a recombineering approach previously developed by Dr. Copeland’s group [23], [24]. From the bMQ323G13 BAC clone (Sanger Institute, Cambridge, UK), we subcloned a 12.8-kb fragment spanning introns 17–21 of *Slc26a4* into the PL253 plasmid (Fig. 1A). The subcloned genomic 12.8-kb region was modified in a subsequent targeting round by inserting the neomycin (*neo*) cassette from the PL452 plasmid and creating the c.2168A>G mutation in exon 19. The targeting vector was then linearized by *Not*I digestion and electroporated into R1 embryonic stem (ES) cells. G418 (240 µg/mL) and ganciclovir (2 µM) double-resistant clones were analyzed by Southern blot hybridization (Fig. 1B). The retained *neo* cassette flanked by *loxP* sites was excised *in vivo* by transfecting the targeted clone with plasmid transiently expressing the Cre recombinase. Established ES clones were then identified by polymerase chain reaction (PCR) screening and subsequently injected into C57BL/6 blastocysts to produce chimeras. After germline transmission of the targeted mutation allele, we produced the congenic *Slc26a4^+/tm2Dontuh^* mouse line used in this study by repeated backcrossing into the C57BL/6 inbred strain for 6–10 generations, after which mice homozygous for the mutation (i.e., *Slc26a4^tm2Dontuh/tm2Dontuh^*) were obtained by intercrossing heterozygous mice (i.e., *Slc26a4^+/tm2Dontuh^*) (Fig. 1C). Reverse transcription-PCR (RT-PCR) of mRNA of inner ear extract followed by direct sequencing also indicated a pure non-chimeric genetic background without unintentionally wild-type *Slc26a4* expression in *Slc26a4^tm2Dontuh/tm2Dontuh^* mice. Corresponding to the human genotypes, mice with compound heterozygous mutations for p.H723R and c.919-2A>G (i.e., *Slc26a4^tm1Dontuh/tm2Dontuh^*) were also generated by intercrossing heterozygous *Slc26a4^+/tm2Dontuh^* mice with *Slc26a4^tm1Dontuh/tm1Dntuh^* mice. All animal experiments were carried out in accordance with animal welfare guidelines and approved by the Institutional Animal Care and Use Committee (IACUC) of National Taiwan University College of Medicine (approval no. 20110123).

### Audiological and Vestibular Evaluations

For audiological evaluations, the mice were anesthetized with sodium pentobarbital (35 mg/kg) delivered intraperitoneally and placed in a head-holder within an acoustically and electrically insulated and grounded test room. We used an evoked potential detection system (Smart EP 3.90; Intelligent Hearing Systems, Miami, FL, USA) to measure the thresholds of the auditory brainstem response (ABR) in mice. Click sounds, as well as 8, 16, and 32 kHz tone bursts at varying intensity, were generated to evoke ABRs in mice. The response signals were recorded with subcutaneous needle electrodes. The active electrodes were inserted into the vertex and the ipsilateral retro-auricular region with a ground electrode on the back of the mice.

For vestibular evaluations, mice were subjected to a battery of tests, including observation of their circling behavior and head tilting (performed at 3 weeks of age), reaching test, swimming test, gripping test, and a rotarod test (all performed at 8 weeks of age). The methodology of each vestibular test is described in details in our previous study [16].

### Inner Ear Morphology Studies

Tissues from the inner ears of mice were subjected to hematoxylin and eosin (H&E) staining, and the morphology of each sample was examined with a Leica optical microscope. For both light microscopy and scanning electron microscopy (SEM) studies, inner ears from adult mice were fixed by perilymphatic perfusion with 4% paraformaldehyde (PFA) in phosphate-buffered saline (PBS) through round and oval windows and a small fenestra in the apex of the cochlear bony capsule. Specimens were subsequently rinsed in PBS buffer and decalcified in 4% PFA with 0.35 M EDTA at 4°C for 1 week. For light microscopy studies, the samples were dehydrated and embedded in paraffin. Subsequently, serial sections (7 µm) were stained with H&E. For SEM studies, the samples were dehydrated in ethanol, critical-point dried, gold sputter coated, and then examined in a field emission scanning electron microscope (S-4500; Hitachi, Tokyo, Japan).

Whole-mount studies of mouse inner ear specimens were performed as previously described [25] with some minor modifications. Briefly, after perfusion with 4% PFA, the cochleae were postfixed in the same solution for 2 h at room temperature and washed in PBS. The samples were permeabilized in 1% Triton X-100 for 30 min and washed with PBS, followed by overnight incubation at 4°C in the blocking solution. The tissues were then stained with rhodamine-phalloidin (1∶100 dilution; Molecular Probes, Eugene, OR, USA). After washing in PBS, the tissues were mounted using the ProLong Antifade kit (Molecular Probes, Eugene, OR, USA) for 20 min at room temperature. Images of the tissues were obtained using a laser scanning confocal microscope (Zeiss LSM 510; Germany).

### Expression of Pendrin

For pendrin expression experiments, we prepared tissue sections from the inner ears of *Slc26a4^tm1Dontuh/tm2Dontuh^* and *Slc26a4^tm2Dontuh/tm2Dontuh^* mice. Tissue sections mounted on silane-coated glass slides were then deparaffinized in xylene and rehydrated in ethanol. After antigen heat retrieval (500 W microwave oven, in 10 mm citric buffer, pH 6.0, for 20 min), the slides were incubated overnight at 4°C with primary antibodies in PBS and Tween (PBST) (rabbit anti-pendrin, 1∶100 [H195]; mouse anti-Myosin VIIa, 1∶100 [C-5]; Santa Cruz Biotechnology, Santa Cruz, CA, USA). The slides were then washed and incubated for 1 h at 25°C with appropriate secondary antibodies at a 1∶1000 dilution in PBST. After incubation, the slides were washed with PBST and mounted with the ProLong Antifade kit at 25°C. Images were obtained using a laser scanning confocal microscope (Zeiss LSM 510; Germany).

### Real-time PCR

Total RNA was purified using Trizol reagent (Invitrogen, CA, USA) according to the manufacturer’s protocol and stored at −80°C until further use. Total RNA was treated with DNase and then reverse transcribed into first-strand cDNA in a 20-µL reaction volume using SuperScript III Reverse Transcriptase (Invitrogen, CA, USA). Quantitative real-time reverse transcription (RT)-PCR assays of *Kcnj10* cDNA were performed using a gene-specific double fluorescently labeled TaqMan probe in an ABI Prism 7900 Sequence Detection System (Applied Biosystems) in accordance with the manufacturer’s recommendations. The relative mRNA expression level was determined by the 2^−ΔΔCt^ analysis method, and calculations were performed using the software provided by the manufacturer (Applied Biosystems). Specimens from 5 mice were collected for each experiment, and the experiments were performed in triplicates and averaged. The average mRNA expression level in wild-type mice was normalized to 1 as the control.

### Western Blot Analysis

Protein extracts of the inner ear were homogenized in RIPA buffer (Millipore, MO, USA). Equal amounts of proteins were supplemented with dithiothreitol, heated for 5 min at 95°C, separated by SDS gel electrophoresis, and then transferred to a PVDF membrane (Amersham Pharmacia Biotech, Little Chalfont, UK) by semi-dry electroblotting. The PVDF membranes were developed with an enhanced chemiluminescence western blot detection kit (Pierce SuperSignal® West Dura, Rockford, IL, USA) and exposed to Lumi-Film chemiluminescent detection films (Roche Diagnostics, Mannheim, Germany). The specimens were collected from 5 mice for each experiment, and the experiments were performed in triplicates and averaged. The average protein expression level in wild-type mice was normalized to 1 as the control.

### Noise Exposure Experiments

For the noise exposure experiments, 10 mice of each genotype were exposed for 3 h to octave-band noise with a peak at 4 kHz, 115 dB SPL. The noise room was fitted with a speaker (model NO. 1700–2002, GSI) driven by a noise generator (GSI-61; Grason-Stadler, Inc.) and a power amplifier (5507-Power; TECHRON). ABR thresholds (dB SPL) in mice at different frequencies (click, 8 kHz, 16 kHz, and 32 kHz) were then recorded at 30 min and at day 1, 2, 3, 7, and 14 after noise exposure.

### Thyroid and Renal Serum Biochemistry

Terminal blood samples were obtained from mice at postnatal day 15 to compare the early postnatal T4 surge [26], as well as at 2 and 6 months of age to monitor the long-term thyroid and renal profiles. Total T4 was measured in undiluted serum (25 µL) by RIA (Diagnostic Products Corp., Los Angeles, CA, USA). Thyroid-stimulating hormone (TSH), Blood urea nitrogen (BUN) and serum creatinine (CREA) were measured by the research services of National Taiwan University Hospital.

## Results

### Audiological and Vestibular Phenotypes

**Figure 2:**
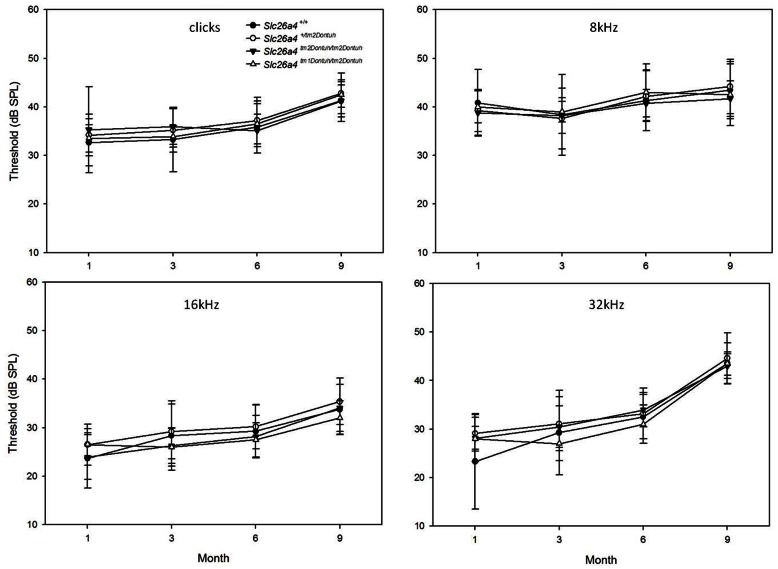
Hearing thresholds (dB SPL) of different frequencies (clicks, 8, 16, and 32 kHz) at 1, 3, 6, and 9 months in mice with different genotypes. Heterozygous mice (i.e., *Slc26a4^+/tm2Dontuh^*), homozygous mice (i.e., *Slc26a4^tm2Dontuh/tm2Dontuh^*), and compound heterozygous mice (i.e., *Slc26a4^tm1Dontuh/tm2Dontuh^*) showed normal hearing as wild type mice (*Slc26a4^+/+^* ) up to 9 months.

Wild-type mice (i.e., *Slc26a4^+/+^*), heterozygous mice (i.e., *Slc26a4^+/tm2Dontuh^*), and homozygous mice (i.e., *Slc26a4^tm2Dontuh/tm2Dontuh^*) (n = 10 each) were subjected to audiological evaluations at 1, 3, 6, and 9 months. Both *Slc26a4^+/tm2Dontuh^* and *Slc26a4^tm2Dontuh/tm2Dontuh^* mice had normal hearing up to 9 months (Fig. 2), indicating that the p.H723R allele does not lead to deafness in mice. To verify the pathogenicity of the p.H723R allele in mice, we further generated mice with compound heterozygous mutations (i.e., *Slc26a4^tm1Dontuh/tm2Dontuh^*) by intercrossing *Slc26a4^+/tm2Dontuh^* mice with *Slc26a4^tm1Dontuh/tm1Dontuh^* mice, which segregated the c.919-2A>G mutation with an abolished function [16]. Similar to the mice heterozygous for the c.919-2A>G mutation (i.e., *Slc26a4^+/tm1Dontuh^*), *Slc26a4^tm1Dontuh/tm2Dontuh^* mice (n = 10) had normal hearing up to 9 months; this finding confirmed that the p.H723R allele was not pathogenic and a single p.H723R allele was sufficient to maintain normal hearing in mice.

A total of 60 mice, including *Slc26a4^+/+^* mice, *Slc26a4^+/tm2Dontuh^* mice, *Slc26a4^tm2Dontuh/tm2Dontuh^* mice, and *Slc26a4^tm1Dontuh/tm2Dontuh^* mice (n = 15 each), were subjected to vestibular evaluations (Table S1). Similar to the normal audiological phenotypes, neither heterozygous mice (i.e., *Slc26a4^+/tm2Dontuh^*) nor homozygous mice (i.e., *Slc26a4^tm2Dontuh/tm2Dontuh^*) showed vestibular deficits such as head tilting and circling behavior, and both groups performed normally on reaching, swimming, gripping, and rotarod tests. Similarly, compound heterozygous mice (i.e., *Slc26a4^tm1Dontuh/tm2Dontuh^*) also did not show vestibular deficits, indicating that a single p.H723R allele was sufficient to maintain normal vestibular function in mice.

### Cochlear Morphology

**Figure 3:**
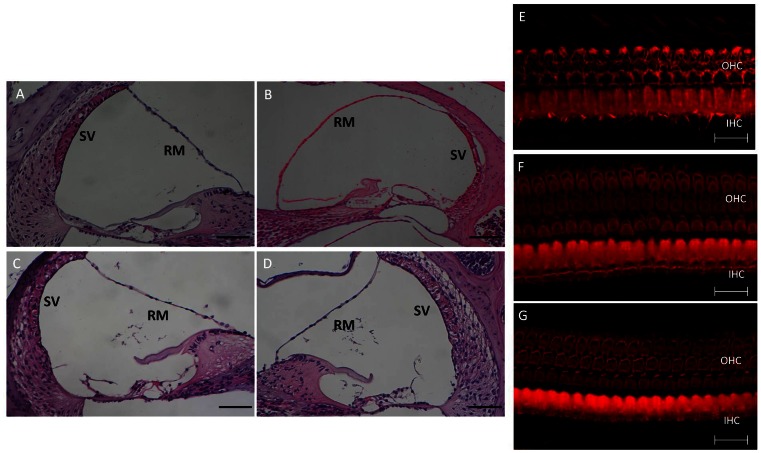
Comparison of Cochlear morphology in mice with different genotypes at P60. Compared with cochlear morphology in *Slc26a4^+/+^* mice (A), severe endolymphatic hydrops (dilatation of scala media) and a significant atrophy of the stria vascularis (B), as well as degenerated hair cells (E), were observed in *Slc26a4^tm1Dontuh/tm1Dontuh^* mice. In contrast, normal cochlear morphology was revealed in both *Slc26a4^tm2Dontuh/tm2Dontuh^* mice (C) and *Slc26a4^tm1Dontuh/tm2Dontuh^* mice (D). No degeneration of cochlear hair cells at the basal turn was observed in *Slc26a4^tm2Dontuh/tm2Dontuh^* mice (F) and *Slc26a4^tm1Dontuh/tm2Dontuh^* mice (G). IHC: inner hair cells; OHC: outer hair cells; RM: Reissner’s membrane; SV: stria vascularis; A, B, C, D: hematoxylin and eosin (H&E) staining; E, F, G: fluorescence confocal microscopy; Bar = 150 µm (A–D) and 20 µm (E–G).

Cochlear morphology was investigated in homozygous mice (i.e., *Slc26a4^tm2Dontuh/tm2Dontuh^*) and compound heterozygous mice (i.e., *Slc26a4^tm1Dontuh/tm2Dontuh^*). The cochlear morphologies of wild-type mice and the profoundly deaf *Slc26a4^tm1Dontuh/tm1Dontuh^* mice were also compared. Abnormal morphological findings in *Slc26a4^tm1Dontuh/tm1Dontuh^* mice, such as severe endolymphatic hydrops with dilatation of the scala media (Fig. 3B), significant atrophy of the stria vascularis (Fig. 3B), and degeneration of the cochlear hair cells (Fig. 3E), were not observed in *Slc26a4^tm2Dontuh/tm2Dontuh^* mice (Fig. 3C and 3F) and *Slc26a4^tm1Dontuh/tm2Dontuh^* mice (Fig. 3D and 3G).

### Vestibular Morphology

**Figure 4:**
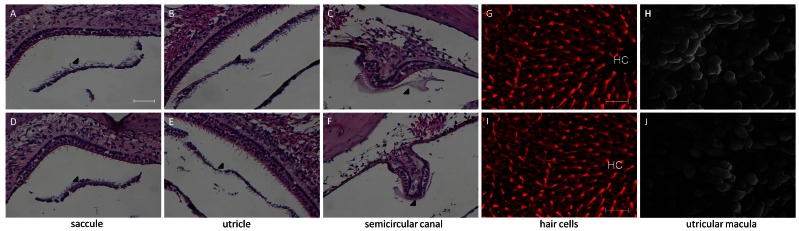
Comparison of Vestibular morphology in mice with different genotypes at P60 (*Slc26a4^tm2Dontuh/tm2Dontuh^* mice: A, B, C, G, H; *Slc26a4^tm1Dontuh/tm2Dontuh^* mice: D, E, F, I, J). Normal morphological findings and amount of otoconia in the vestibule (arrowhead) are shown in both mice (A, B, C, D, E, F). No degeneration of vestibular hair cells in both mice is observed by fluorescence confocal microscopy (G, H). Scanning electron microscopic findings show normal otoconia at the utricle in both mice (I, J). Bar = 50 µm (A–F) and 10 µm (G–H).

The vestibular morphology was investigated in homozygous mice (i.e., *Slc26a4^tm2Dontuh/tm2Dontuh^*) (Fig. 4A, 4B, and 4C) and compound heterozygous mice (i.e., *Slc26a4^tm1Dontuh/tm2Dontuh^*) (Fig. 4D, 4E, and 4F). Both mice showed normal morphological findings and amount of otoconia in the vestibule. Fluorescence confocal microscopy revealed that vestibular hair cells in *Slc26a4^tm2Dontuh/tm2Dontuh^* mice and *Slc26a4^tm1Dontuh/tm2Dontuh^* mice were not degenerated (Fig. 4G and 4I). SEM revealed normal otoconia at the utricle in *Slc26a4^tm2Dontuh/tm2Dontuh^* mice and *Slc26a4^tm1Dontuh/tm2Dontuh^* mice (Fig. 4H and 4J).

### Immunolocalization and Expression of Pendrin

**Figure 5:**
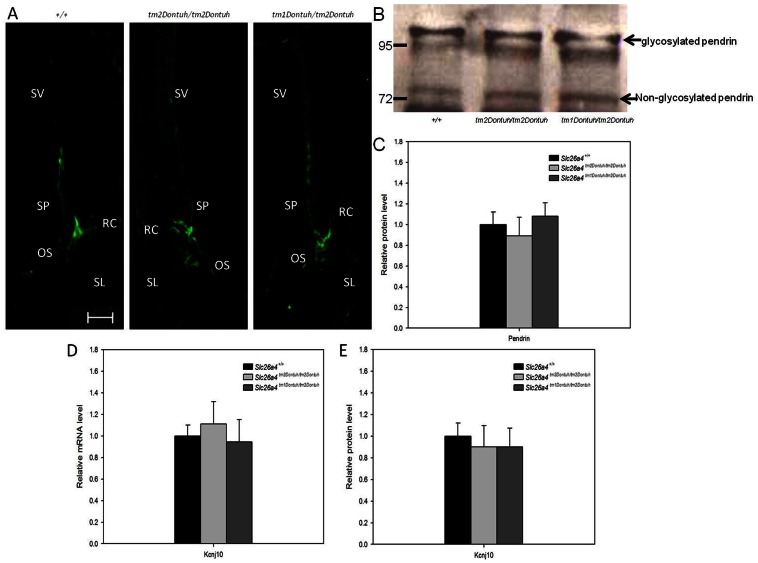
Expression of pendrin and *Kcnj10*. (A) Pendrin is normally distributed in the spiral prominence and root cells (stained in green) in *Slc26a4^+/+^* (left), *Slc26a4^tm2Dontuh/tm2Dontuh^* (middle), and *Slc26a4^tm1Dontuh/tm2Dontuh^* mice (right), indicating that the expression of pendrin is not affected by the p.H723R mutation in mice. (B) Immunoblotting of pendrin expression at P42. Both *Slc26a4^tm2Dontuh/tm2Dontuh^* and *Slc26a4^tm1Dontuh/tm2Dontuh^* mice expressed pendrin of molecular weight comparable to the wild-type mice, indicating that the glycosylation process remained unaffected in the p.H723R-pendrin. (C) Quantification of pendrin protein expression at P42. The expression levels of pendrin in *Slc26a4^tm2Dontuh/tm2Dontuh^* mice and *Slc26a4^tm1Dontuh/tm2Dontuh^* mice were 0.89±0.18 and 1.08±0.13, showing no significant difference as compared with 1.00±0.11 in *Slc26a4^+/+^* mice (mean percentage ± SE, n = 3). (D) Quantification of mRNA expression of *Kcnj10* at P15 by real-time PCR. *Slc26a4^tm2Dontuh/tm2Dontuh^* mice and *Slc26a4^tm1Dontuh/tm2Dontuh^* mice did not show significantly different mRNA levels of *Kcnj10* as compared with *Slc26a4^+/+^* mice. (E) Quantification of Kcnj10 protein expression at P15 by western blotting. The expression levels of Kcnj10 protein in *Slc26a4^tm2Dontuh/tm2Dontuh^* mice and *Slc26a4^tm1Dontuh/tm2Dontuh^* mice were 0.90±0.19 and 0.90±0.17, showing no significant difference as compared with 1.00±0.12 in *Slc26a4^+/+^* mice (mean percentage ± SE, n = 3). RC: root cells; SP: spiral prominence; SL: spiral ligament; SV: stria vascularis; Bar = 20 µm.

We then investigated the expression of pendrin in the cochlea of *Slc26a4^tm2Dontuh/tm2Dontuh^* mice and *Slc26a4^tm1Dontuh/tm2Dontuh^* mice (Fig. 5A) by immunolocalization. In both strains of mice, pendrin was normally distributed in the spiral prominence and root cells, indicating that the expression of pendrin was not affected by the p.H723R mutation in mice. Compared with the wild-type mice, no significant difference in the molecular weight (Fig. 5B) or the expression level (Fig. 5C) of pendrin were observed in *Slc26a4^tm2Dontuh/tm2Dontuh^* mice and *Slc26a4^tm1Dontuh/tm2Dontuh^* mice by western blotting analyses.

### Kcnj10 Expression

We then investigated the expression of *Kcnj10* (GeneID: 16513) in *Slc26a4^+/+^*mice, *Slc26a4^tm2Dontuh/tm2Dontuh^* mice, and *Slc26a4^tm1Dontuh/tm2Dontuh^* mice by real-time PCR (for mRNA expression) and western blotting (for protein expression) analyses. It has been demonstrated that_*Slc26a4*-depleted mice showed decreased *Kcnj10* expression, which contributes to the failure of endocochlear potential generation [27]. *KCNJ10* (GeneID: 3766) mutations have been observed in EVA patients via digenic inheritance with *SLC26A4* mutations [28]. In this study, the stria vascularis of P15 mouse cochleae from *Slc26a4* ^+/+^ mice, *Slc26a4^tm2Dontuh/tm2Dontuh^* mice, and *Slc26a4^tm1Dontuh/tm2Dontuh^* mice were isolated by microdissection, and total RNA and protein extracted from these tissue fractions were used for real-time PCR and quantitative immunoblot analyses. Compared with the wild-type mice, no significant differences in the mRNA (Fig. 5D) or protein levels of Kcnj10 (Fig. 5E) were observed in *Slc26a4^tm2Dontuh/tm2Dontuh^* mice and *Slc26a4^tm1Dontuh/tm2Dontuh^* mice.

### Noise Exposure Experiments

**Figure 6:**
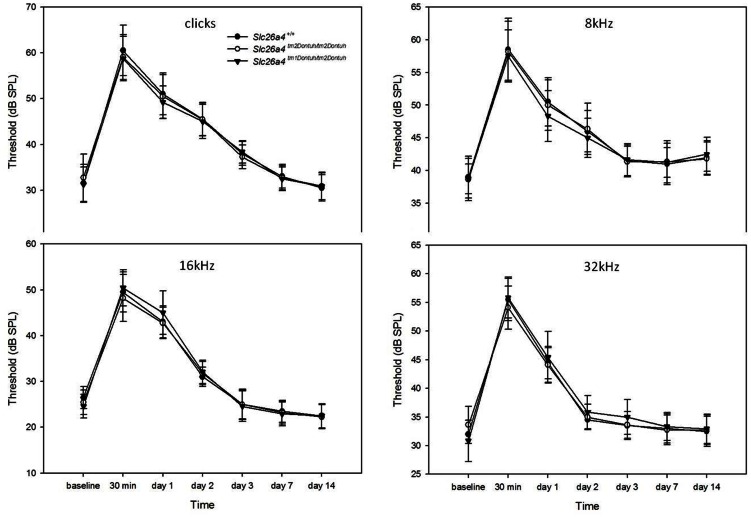
Chronological change of hearing thresholds (dB SPL) of different frequencies (clicks, 8, 16, and 32 kHz) in mice with different genotypes. Neither *Slc26a4^tm2Dontuh/tm2Dontuh^* mice nor *Slc26a4^tm1Dontuh/tm2Dontuh^* mice had a significantly higher shift in hearing thresholds at any of the frequencies at 30 min or at day 1, 2, 3, 7, and 14 after noise exposure as compared with *Slc26a4^+/+^* mice.

We then attempted to induce audiologic phenotypes in transgenic mice with noise exposure [29]. Neither *Slc26a4^tm2Dontuh/tm2Dontuh^* mice nor *Slc26a4^tm1Dontuh/tm2Dontuh^* mice showed a significantly higher shift in hearing thresholds at all frequencies 30 min and at day 1, 2, 3, 7, and 14 after noise exposure than *Slc26a4^+/+^* mice (Fig. 6); this finding confirmed their normal audiologic phenotypes.

### Thyroid and Renal Profiles

Goiter was not observed in *Slc26a4^+/+^* mice, *Slc26a4^tm2Dontuh/tm2Dontuh^* mice, and *Slc26a4^tm1Dontuh/tm2Dontuh^* mice (n = 5 each) until the mice were 6-months old. Blood chemistry, including total T4, TSH, BUN, and CREA, were all within normal limits at postnatal day 15, 2 and 6 months (Table S2).

## Discussion

In this study, we generated a knock-in mouse model, denoted as *Slc26a4^tm2Dontuh/tm2Dontuh^* mice, which segregates the common deafness-associated p.H723R mutation in humans. The p.H723R mutation was found in both nonsyndromic EVA and PS families [18], [30]. This missense mutation was the most prevalent *SLC26A4* mutation among the Japanese [18] and Koreans [19], and the second most prevalent *SLC26A4* mutation among the Han Chinese [20], [21]. Recent reports indicated that the p.H723R mutation arose from a common ancestor and was not a mutational hot spot [31]. The pathogenicity of the p.H723R mutation in humans was confirmed by several lines of evidence supporting the fact that p.H723R co-segregated with the phenotypes in the affected families, the prevalence of p.H723R was low in the control populations, and the p.H723 amino acid residue was evolutionally conserved [18]. Moreover, in human cell lines, the p.H723R has been related to trafficking/folding/glycosylation defects of the pendrin protein. In transfected HEK293 cells and HeLa cells, p.H723R caused protein retention in endoplasmic reticulum and abolished complex glycosylation of pendrin, and the defects in protein processing could be restored considerably by low temperature incubation or treatment with sodium butyrate [32]. Further study revealed the folding defect in the p.H723R-pendrin, and treatment of salicylate, which functioned as a pharmacological chaperone, could restore normal protein localization and anion exchange activity [33].

Interestingly, as shown in the present study, both heterozygous mice (i.e., *Slc26a4^+/tm2Dontuh^*) and homozygous mice (i.e., *Slc26a4^tm2Dontuh/tm2Dontuh^*) with the knock-in p.H723R mutation had normal audiovestibular phenotypes, including normal hearing thresholds up to 9 months, excellent balancing ability, and an intact cochlear and vestibular morphology. There are several possibilities why the p.H723R mutation did not lead to the expected abnormal phenotype in mice. The first possibility is that the abnormal phenotype was present but not yet discovered or that the abnormal phenotype will become evident only under certain environmental conditions [34]. Although this possibility could not be completely ruled out by the results of this study, it was largely weakened after an exhaustive investigation of the audiovestibular phenotypes, including examination of hearing levels up to 9 months, a battery of vestibular tests, comprehensive inner ear morphological studies, and noise exposure experiments. The second possibility is that the phenotypic effects of the p.H723R mutation are influenced by the genetic background of the mice [35]. This was also unlikely; as in our previous study, we generated a knock-in *Slc26a4* mouse model with abnormal audiovestibular phenotypes by using the same C57BL/6 strain. Another possibility, which became the most likely scenario for our study after the former 2 possibilities were refuted, is that, there are, in fact, no abnormal phenotypes.

In particular, the absence of abnormal audiovestibular phenotypes in homozygous mice alone is not sufficient to exclude the pathogenicity of p.H723R in mice because the function of the *Slc26a4* p.H723R mutation might be only partially defective, and even harboring 2 p.H723R alleles might still maintain adequate *Slc26a4* function. To elucidate the pathogenicity of the p.H723R allele, we further generated mice with compound heterozygous mutations (i.e., *Slc26a4^tm1Dontuh/tm2Dontuh^*) by intercrossing *Slc26a4^+/tm2Dontuh^* mice with *Slc26a4^tm1Dontuh/tm1Dontuh^* mice, which segregated the c.919-2A>G mutation with abolished function. Mice that were compound heterozygous for p.H723R and c.919-2A>G (i.e., *Slc26a4^tm1Dontuh/tm2Dontuh^*) had normal audiovestibular phenotypes, indicating that a single p.H723R allele was sufficient to maintain normal inner ear physiology. Moreover, the expression of the downstream *Kcnj10* gene, which diminished at P10–P15 following oxidative stress in *Slc26a4^−/−^* mice [36], was not compromised in *Slc26a4^tm2Dontuh/tm2Dontuh^* mice and *Slc26a4^tm1Dontuh/tm2Dontuh^* mice. These findings indicate that the p.H723R allele is neither pathogenic in mice nor is a mutation with partially ablated function.

As shown in Figure 5, both *Slc26a4^tm2Dontuh/tm2Dontuh^* and *Slc26a4^tm1Dontuh/tm2Dontuh^* mice expressed pendrin of molecular weight and expression level comparable to the wild-type mice, indicating that the trafficking/glycosylation process remained unaffected in the murine p.H723R-pendrin. The difference in the intactness of the trafficking/glycosylation process might contribute to the variation in the pathogenicity of p.H723R between mice and humans. We further analyzed the amino-acid sequences of human and mouse pendrin using ConSeq (http://conseq.tau.ac.il/), a website server predicting biologically important residues in protein [37]. Possibly the intact trafficking/glycosylation process of p.H723R in mice might be attributed to a different alignment of amino acid residues in the vicinity, as well as the embedded location of p.H723 in the pendrin (Fig. S1).

Four other mouse models with *Slc26a4* mutations have been reported: knock-out *Slc26a4^−/−^* mice [14], *Slc26a4^loop^* mice with the p.S408F mutation generated by ENU mutagenesis [15], *Slc26a4^tm1Dontuh^* knock-in mice with the common East Asian c.919-2A>G mutation [16], and conditional knock-out Tg[E];Tg[R];*Slc26a4* ^Δ*/*Δ^ mice [17] (Table 1). *Slc26a4^−/−^* mice are profoundly deaf with significant vestibular deficits and have EVA and scala media, mimicking the phenotypes in humans [14]. Subsequent research on *Slc26a4^loop^* mice [15] and *Slc26a4^tm1Dontuh/tm1Dontuh^* mice [16] reported similar auditory and vestibular characteristics, complementing the studies on *Slc26a4^−/−^* mice. To better simulate the less severe audiological phenotype in humans, Choi et al. generated a binary transgenic mouse line in which *Slc26a4* expression could be induced with doxycycline. The authors identified that the E16.5 to P2 was the critical interval in which pendrin was required for acquisition of normal hearing, and demonstrated that doxycycline initiation at E18.5 or discontinuation at E17.5 resulted in partial hearing loss, instead of the profound hearing loss observed in the knock-out *Slc26a4^−/−^* mice [17]. In contrast, *Slc26a4^tm2Dontuh/tm2Dontuh^* mice examined in this study had normal audiovestibular phenotypes, representing a distinct and unique mouse strain compared with the other mouse models reported in the literature.

**Table 1:** Comparison of phenotypes among mouse strains segregating different Slc26a4 mutations.

	*Slc26a4^−/−^*	*Slc26a4^loop/loop^*	*Slc26a4^tm1Dontuh/tm1Dontuh^*	Tg[E];Tg[R];*Slc26a4* ^Δ*/*Δ^	*Slc26a4^tm2Dontuh/tm2Dontuh^*
Audiological phenotypes	Profound hearing loss (>100 dB SPL)	Profound hearing loss(>100 dB SPL)	Profound hearing loss(>120 dB SPL)	Hearing levels dependon the time of *Slc26a4*expression. Doxycyclineinitiation at E18.5 (IE 18.5)results in partialhearing loss.	Normal
Vestibular phenotypes	Vestibular deficits,including head-tiltingand circling	Variable vestibular deficits,including circling andtilted body.	46% of mice withhead-tilting and circling.	ND	Normal
Inner Ear Morphology Vestibular aqueduct	Enlarged	ND	Enlarged	Sizes depend on the timeof *Slc26a4* expression.Significantly enlargedin IE18.5 mice.	Normal
Scala media	Enlarged	ND	Enlarged	Not enlarged (IE18.5)	Normal
Cochlear hair cells	Severe degenerationof inner and outerhair cells by P30.	ND	Severe degenerationof inner and outer haircells at 6 wks.	Functionally intact atP25 to P35 (IE18.5)	Normal
Vestibular hair cells	Severe degenerationof vestibular haircells by P30.	Normal morphologyof vestibular hair cellsat 2 m.	Loss and degeneration ofutricular hair cellscorrelated to thevestibular phenotypes	ND	Normal
Otoconia	Almost completeabsence of otoconiawith occasionalpresence ofgiant otoconia.	Giant otoconia in the utriclefrom P0 to 10 m; gradualchange in otoconiacomposition to calciumoxalate in the saccule fromP0 to 10 m; ectopicotoconia in thesemicircular canal.	Decreased amount ofotoconia in the sacculeand utricle, formationof giant otoconia in thesaccule and utricle, andectopic distribution ofotoconia into thesemicircular canal	ND	Normal

ND, not described.

*Slc26a4^loop/loop^*: mice homozygous for the p.S408F mutation.

Among humans, patients with PS are more likely to have 2 *SLC26A4* mutant alleles than those with nonsyndromic EVA [11], [12]. Moreover, the number of *SLC26A4* mutant alleles is significantly correlated with the severity of hearing loss in individuals with EVA [38], [39]. However, because of its diverse mutation spectrum, it is difficult to delineate the phenotypes associated with a specific *SLC26A4* mutation in humans. Consequently, determining the pathogenetic mechanisms of each specific *SLC26A4* mutation largely relies on functional studies performed in *Xenopus* oocytes [40] or cell lines [41], [42]. The observation that mice with distinct *Slc26a4* mutations have different phenotypes indicates that transgenic mice may serve as appropriate, direct models to investigate corresponding *SLC26A4* mutations in humans.

In conclusion, using a genotype-driven approach, we generated a knock-in mouse model segregating the common deafness-associated *SLC26A4* p.H723R mutation in humans. To our surprise, mice with the *Slc26a4* p.H723R mutation had a normal audiovestibular phenotype and inner ear morphology. Because there might be differences in the pathogenicity of specific *SLC26A4* mutations in humans and mice, precaution should be taken when extrapolating the results of animal studies to humans.

## Supporting Information

Figure S1 **Alignment of amino-acid sequences of human and mouse pendrin.** The amino-acid sequence (a.a. 651–780) of human pendrin (hum-pendrin) was aligned in relative to the sequence of the mouse pendrin (mse-pendrin) using Conseq. Arrows indicate the p.H723 position. The p.H723 is a highly conserved but buried amino acid residue. Different alignments of amino acid residues in the vicinity of p.H723 and the embedded location of p.H723 in the pendrin might contribute to the variation in the pathogenicity of p.H723R between mice and humans. The first row below the sequence lists the predicted burial status of the site (b, buried; e, exposed). The second row indicates residues predicted to be structurally (s) and functionally (f) important.
(TIF)

Table S1 **Comparison of vestibular features according to the genotypes and the circling behavior.**
(DOCX)

Table S2 **Blood chemistry of** ***Slc26a4*** **male mice at postnatal day 15, 2 and 6 months of age.**
(DOCX)
