# Supplementary figures and images for: Differences in the Pathogenicity of the p.H723R Mutation of the Common Deafness-Associated SLC26A4 Gene in Humans and Mice
Source: PLoS One. 2013 Jun 3;8(6):e64906. doi: 10.1371/journal.pone.0064906 (PMC3670936; doi:10.1371/journal.pone.0064906)

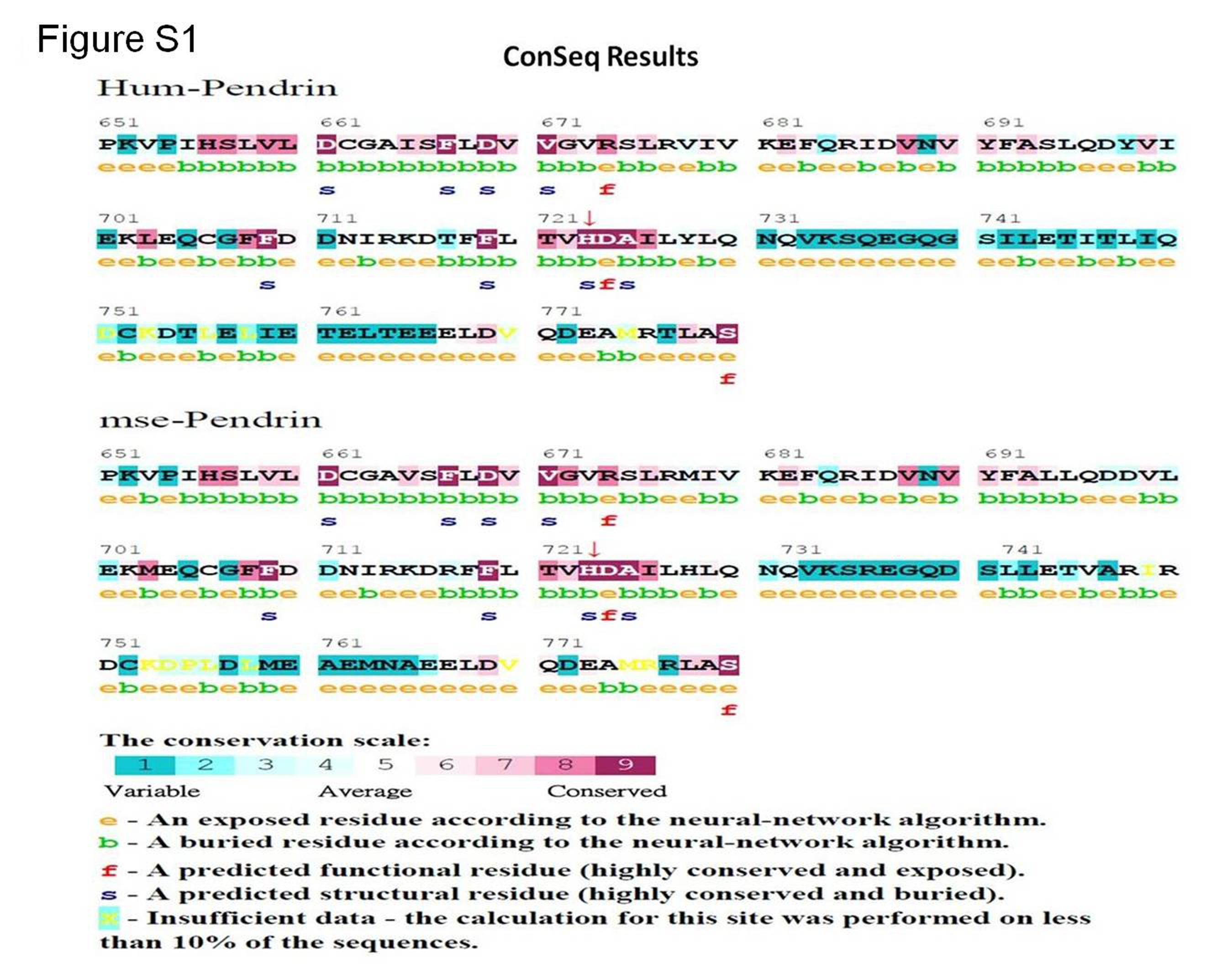

Supplement: Figure S1 — Alignment of amino-acid sequences of human and mouse pendrin. The amino-acid sequence (a.a. 651–780) of human pendrin (hum-pendrin) was aligned in relative to the sequence of the mouse pendrin (mse-pendrin) using Conseq. Arrows indicate the p.H723 position. The p.H723 is a highly conserved but buried amino acid residue. Different alignments of amino acid residues in the vicinity of p.H723 and the embedded location of p.H723 in the pendrin might contribute to the variation in the pathogenicity of p.H723R between mice and humans. The first row below the sequence lists the predicted burial status of the site (b, buried; e, exposed). The second row indicates residues predicted to be structurally (s) and functionally (f) important. (TIF) [file pone.0064906.s001.tif]
