# Supplementary material for: Differences in the Pathogenicity of the p.H723R Mutation of the Common Deafness-Associated SLC26A4 Gene in Humans and Mice
Source: PLoS One. 2013 Jun 3;8(6):e64906. doi: 10.1371/journal.pone.0064906 (PMC3670936; doi:10.1371/journal.pone.0064906)
Supplement: Table S1 — Comparison of vestibular features according to the genotypes and the circling behavior. (DOCX) [file pone.0064906.s002.docx]

**Table S1.** Comparison of vestibular features according to the genotypes and the circling behavior

|  | *Slc26a4^+/+^*  (n=15) | *Slc26a4^+/tm2Dontuh^*  (n=15) | *Slc26a4^tm2Dontuh/tm2Dontuh^*  (n=15) | *Slc26a4^tm1Dontuh/tm2Dontuh^*  (n=15) |
| --- | --- | --- | --- | --- |
| Head tilting* (n, abnormal/total) | 0/15 | 0/15 | 0/15 | 0/15 |
| Reaching** (n, abnormal/total) | 0/15 | 0/15 | 0/15 | 0/15 |
| Swimming** (n, abnormal/total) | 0/15 | 0/15 | 0/15 | 0/15 |
| Gripping** (n, abnormal/total) | 0/15 | 0/15 | 0/15 | 0/15 |
| Time on rod** (sec) | 161.7 ± 12.6 | 160.3 ± 18.1 | 156.3 ± 10.4 | 154.0 ± 9.6 |

* Observed at 3 weeks.

** Tested at 8 weeks.
