# Supplementary material for: Differences in the Pathogenicity of the p.H723R Mutation of the Common Deafness-Associated SLC26A4 Gene in Humans and Mice
Source: PLoS One. 2013 Jun 3;8(6):e64906. doi: 10.1371/journal.pone.0064906 (PMC3670936; doi:10.1371/journal.pone.0064906)
Supplement: Table S2 — Blood chemistry of Slc26a4 male mice at postnatal day 15, 2 and 6 months of age. (DOCX) [file pone.0064906.s003.docx]

**Table S2.** Blood chemistry of *Slc26a4* male mice at postnatal day 15, 2 and 6 months of age

|  | *Slc26a4^+/+^*  (n=5) | *Slc26a4^tm2Dontuh/tm2Dontuh^* (n=5) | *Slc26a4^tm1Dontuh/tm2Dontuh^* (n=5) |
| --- | --- | --- | --- |
| P15  BUN (mg/dL)  Creatinine (mg/dL)  Total T4 (μg/dL)  TSH (ng/mL) | 32±4  <0.2  4.1±0.2  4.4±0.1 | 33±4  <0.2  4.2±0.2  4.3±0.1 | 34±4  <0.2  4.1±0.3  4.4±0.2 |
| 2- month-old  BUN (mg/dL)  Creatinine (mg/dL) | 31±4  <0.2 | 33±4  <0.2 | 35±3  <0.2 |
| Total T4 (μg/dL)  TSH (ng/mL) | 3.3±0.1  4.2±0.1 | 3.2±0.2  4.1±0.2 | 3.2±0.1  4.2±0.2 |
| 6- month-old  BUN (mg/dL) | 34±6 | 35±4 | 34±3 |
| Creatinine (mg/dL) | <0.2 | <0.2 | <0.2 |
| Total T4 (μg/dL) | 3.3±0.2 | 3.2±0.2 | 3.1±0.2 |
| TSH (ng/mL) | 4.1±0.2 | 4.1±0.3 | 4.2±0.4 |
